# Draft Genome Sequence of *Rhizobium* sp. Strain 32-5/1, Isolated from Vicia cracca L. Root Nodules in the Russian Arctic

**DOI:** 10.1128/mra.00287-23

**Published:** 2023-05-11

**Authors:** Polina Guro, Denis Karlov, Irina Kuznetsova, Anna Sazanova, Andrey Belimov, Vera Safronova

**Affiliations:** a All-Russia Research Institute for Agricultural Microbiology (ARRIAM), St. Petersburg, Russian Federation; DOE Joint Genome Institute

## Abstract

This study reports the whole-genome sequence of an endosymbiotic bacterium, *Rhizobium* sp. strain 32-5/1, isolated from root nodules of the legume Vicia cracca L. in the Arctic region of Russia. The genome consists of two plasmids and one chromosome, with a total length of 5,621,108 bp and 59.5% GC content.

## ANNOUNCEMENT

Global climate change is followed by the active movement of plant communities to the north, filling new ecological niches (1). In these areas, pasture phytocenoses can form, a significant part of which are legumes that enter into symbiotic relationships with nitrogen-fixing nodule bacteria. Such pasture phytocenoses are extremely important for herbivore nutrition in the Arctic, especially reindeer and muskoxen (2). The legume Vicia cracca is a promising fodder plant in Arctic phytocenoses and currently has an extensive circumpolar habitat (3). Arctic plant ecosystems provide unique opportunities to study the response of legume-rhizobial symbiosis to climate change.

Nodules of *V. cracca* were collected in the Tiksi settlement (1 August 2021; 71.38302°N, 128.52411°E), situated on the Arctic Ocean coast (Sakha Republic, Russia). Individual root nodules were surface sterilized with 70% ethanol for 1 min, washed thoroughly with sterile tap water, and homogenized. Rhizobium strain 32-5/1 was obtained by plating 100 μL of the homogenized nodules on a yeast extract-maltose-sucrose agar (YMSA) plate. After 6 days of incubation at 28°C, colonies of strain 32-5/1 appeared. Further restreaking of the individual colony was performed for isolation of a pure culture. The pure culture of strain 32-5/1 was then picked and grown overnight in Reasoner’s 2A (R2A) broth at 28°C. Genomic DNA was extracted using the DNeasy blood and tissue kit (Qiagen, Germany) according to the manufacturer’s recommendations. Libraries of genomic DNA were prepared using the ligation sequencing kit (SQK-LSK109) and the native barcoding expansion 1-12 kit (EXP-NBD104; ONT, UK), skipping the DNA-shearing step, sequenced using a MinION sequencer with an R.9.4.1 flow cell (ONT), and then base called using Guppy v5.0.11 with the high-accuracy model (ONT). Quality control of the raw data was performed using NanoStat v1.6.0 and resulted in 117,729 reads, with an average read length of 10,507 bp and an average quality of 11.4 (4). The draft genome sequence was obtained using the Flye v2.9 assembler; default parameters were used for all software unless otherwise noted (5). A circular genome was obtained using Circlator v1.5.5, without a specific starting point for the assembled contigs (6). The genomic statistics were measured using QUAST v5.0.2 (7). Three circular contigs—one chromosome with a length of 4,269,414 bp and two plasmids of 1,086,621 bp and 265,073 bp—were assembled, with a total size of 5.6 Mbp, a GC content of 59.5%, and a mean coverage of ~219×. The genome sequence was annotated using NCBI’s PGAP v6.4 software (8). A total of 5,649 genes were predicted in the genome, including 4,489 protein coding genes and 62 RNA genes (9 rRNAs, 49 tRNAs, and 4 noncoding RNAs [ncRNAs]).

**FIG 1:**
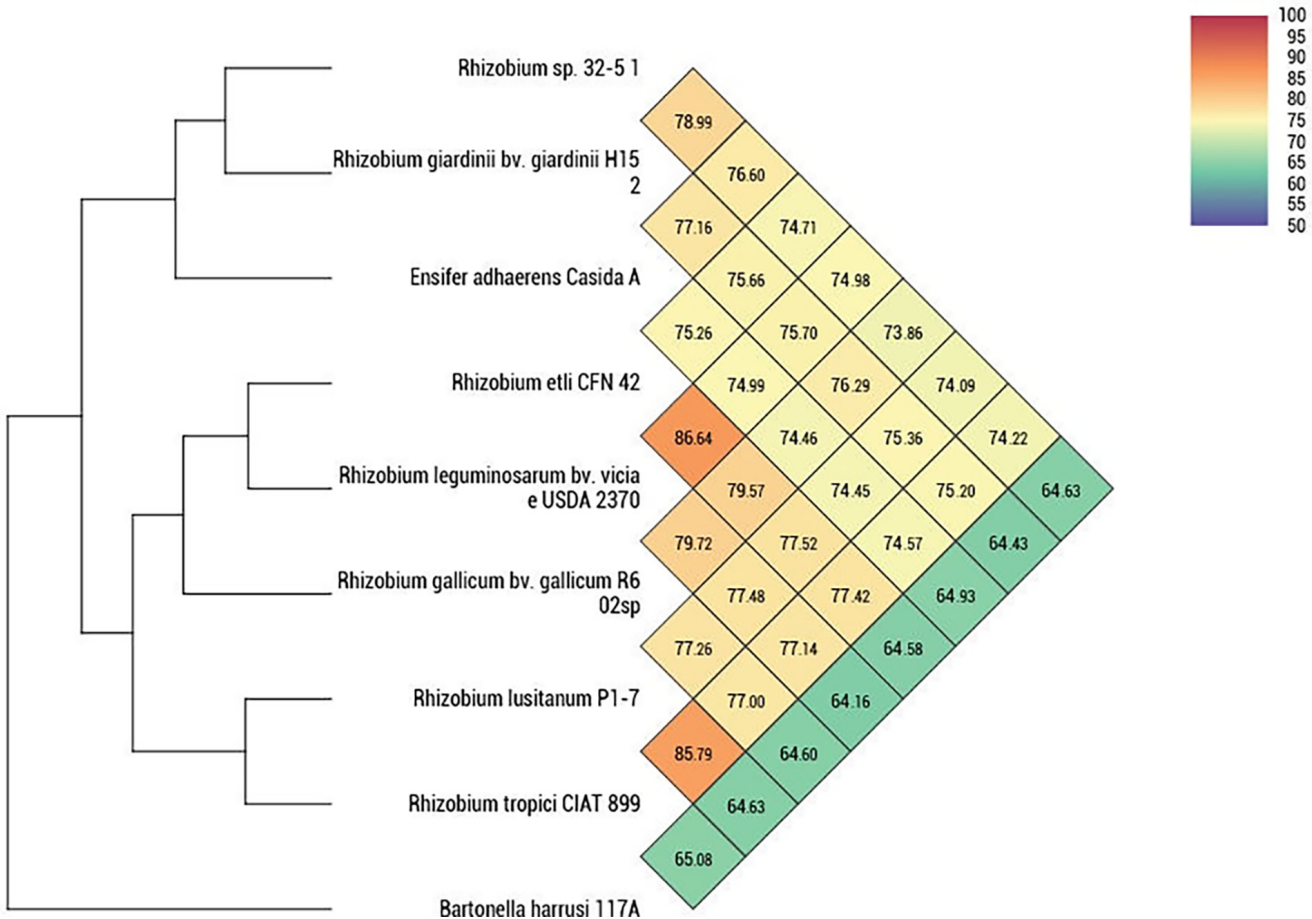
Phylogenetic tree and heat map of orthologous average nucleotide identity (OrthoANI) between strain 32-5/1 and related type strains from the order *Hyphomicrobiales* (genera *Rhizobium* and *Ensifer*), calculated using OAT software, with *Bartonella harrusi* 117A (GenBank accession number NZ_CP101114.1) used as the outgroup.

The taxonomic rank was established using a 16S rRNA (*rrs*) BLASTn comparison, and the average nucleotide identity (ANI) was calculated using OrthoANI in OAT v0.93.1 software (9, 10). Strain 32-5/1 was most closely related to the type strain Rhizobium giardinii H152 (GenBank accession number GCF_000379605.1), with 78.99% average nucleotide identity and 98.11% *rrs* identity (Fig. 1). Based on the OrthoANI and 16S rRNA BLASTn results, strain 32-5/1 was identified as *Rhizobium* sp., without species definition.

### Data availability.

All data are available at GenBank under assembly accession numbers CP120638 to CP120640 and BioProject accession number PRJNA881974. The BioSample accession number is SAMN32638802. The raw MinION data can be found under SRA accession number SRR23086701.

## References

[1] Bjorkman AD, García Criado M, Myers-Smith IH, Ravolainen V, Jónsdóttir IS, Westergaard KB, Lawler JP, Aronsson M, Bennett B, Gardfjell H, Heiðmarsson S, Stewart L, Normand S. 2020. Status and trends in Arctic vegetation: evidence from experimental warming and long-term monitoring. Ambio 49:678–692. doi:10.1007/s13280-019-01161-6. 30929249 PMC6989703

[2] Ustinova VV, Rumyantseva TD. 2022. Deer pasture productivity and crop yield in Anabarsky Ulus of the republic of Sakha (Yakutia). IOP Conf Ser Earth Environ Sci 988:022084. doi:10.1088/1755-1315/988/2/022084.

[3] Wasowicz P, Sennikov AN, Westergaard KB, Spellman K, Carlson M, Gillespie LJ, Saarela JM, Seefeldt SS, Bennett B, Bay C, Ickert-Bond S, Väre H. 2020. Non-native vascular flora of the Arctic: taxonomic richness, distribution and pathways. Ambio 49:693–703. doi:10.1007/s13280-019-01296-6. 31792797 PMC6989699

[4] De Coster W, D'Hert S, Schultz DT, Cruts M, Van Broeckhoven C. 2018. NanoPack: visualizing and processing long-read sequencing data. Bioinformatics 34:2666–2669. doi:10.1093/bioinformatics/bty149. 29547981 PMC6061794

[5] Kolmogorov M, Yuan J, Lin Y, Pevzner PA. 2019. Assembly of long, error-prone reads using repeat graphs. Nat Biotechnol 37:540–546. doi:10.1038/s41587-019-0072-8. 30936562

[6] Hunt M, Silva ND, Otto TD, Parkhill J, Keane JA, Harris SR. 2015. Circlator: automated circularization of genome assemblies using long sequencing reads. Genome Biol 16:294. doi:10.1186/s13059-015-0849-0. 26714481 PMC4699355

[7] Gurevich A, Saveliev V, Vyahhi N, Tesler G. 2013. QUAST: quality assessment tool for genome assemblies. Bioinformatics 29:1072–1075. doi:10.1093/bioinformatics/btt086. 23422339 PMC3624806

[8] Tatusova T, DiCuccio M, Badretdin A, Chetvernin V, Nawrocki EP, Zaslavsky L, Lomsadze A, Pruitt KD, Borodovsky M, Ostell J. 2016. NCBI Prokaryotic Genome Annotation Pipeline. Nucleic Acids Res 44:6614–6624. doi:10.1093/nar/gkw569. 27342282 PMC5001611

[9] Altschul SF, Gish W, Miller W, Myers EW, Lipman DJ. 1990. Basic local alignment search tool. J Mol Biol 215:403–410. doi:10.1016/S0022-2836(05)80360-2. 2231712

[10] Lee I, Kim YO, Park SC, Chun J. 2016. OrthoANI: an improved algorithm and software for calculating average nucleotide identity. Int J Syst Evol Microbiol 66:1100–1103. doi:10.1099/ijsem.0.000760. 26585518

